# Worldwide Increasing Incidences of Cutaneous Malignant Melanoma

**DOI:** 10.1155/2011/858425

**Published:** 2011-10-10

**Authors:** Dianne E. Godar

**Affiliations:** Center for Devices and Radiological Health, Food and Drug Administration, 10903 New Hampshire Avenue, WO64-4024, Silver Spring, MD 20993, USA

## Abstract

The incidence of cutaneous malignant melanoma (CMM) has been increasing at a steady rate in fair-skinned populations around the world for decades. Scientists are not certain why CMM has been steadily increasing, but strong, intermittent UVB (290–320 nm) exposures, especially sunburn episodes, probably initiate, CMM, while UVA (321–400 nm) passing through glass windows in offices and cars probably promotes it. The CMM incidence may be increasing at an exponential rate around the world, but it definitely decreases with increasing latitude up to *~*50°N where it reverses and increases with the increasing latitude. The inversion in the incidence of CMM may occur because there is more UVA relative to UVB for most of the year at higher latitudes. If windows, allowing UVA to enter our indoor-working environment and cars, are at least partly responsible for the increasing incidence of CMM, then UV filters can be applied to reduce the rate of increase worldwide.

## 1. Introduction

The incidence of cutaneous malignant melanoma (CMM) has been increasing at a steady rate in fair-skinned populations around the world for decades [1–12]. Scientists are not certain why CMM has been steadily increasing over the decades, but strong intermittent UVB (290–320 nm) exposures, especially sunburn episodes, evidently initiates CMM [13]. The UVA (321–400 nm) passing through glass windows in offices and cars has been proposed to promote CMM [14]. In support of those possibilities exists the paradox between indoor and outdoor worker's UV exposures and their incidences of CMM. Although outdoor workers get three to ten times the annual UV dose that indoor workers get [15, 16], they have similar or lower incidences of CMM [17]. Scientists think the increasing incidence of CMM is linear based on surveillance epidemiology and end results (SEER) data in the USA that only dates back to 1973 [12], but it may actually be exponential in the USA and in some other regions of the world.

To understand what factor(s) may be responsible for the increasing incidence of CMM, one must know the temporal incidence for as many decades as possible. Because whatever the causative agent is, or agents are, it must have entered or left our environment some time (*∼*10–30 yrs) before the increasing trend was first documented back in 1935 [1]. Thus, one can analyze the CMM incidence data for fair-skinned people around the world and plot it temporally by each country in the northern and southern hemispheres and by latitude. Further analysis of *R* ^2^ values can determine if the curves are linear or exponential. This paper will analyze the CMM incidences of fair-skinned populations all over the world, test if the increases are exponential or linear, and show that the increasing incidence decreases with increasing latitude until *∼*50°N, where it reverses and begins to increase with increasing latitude in Northern Europe.

## 2. Materials and Methods

**Figure 1:**
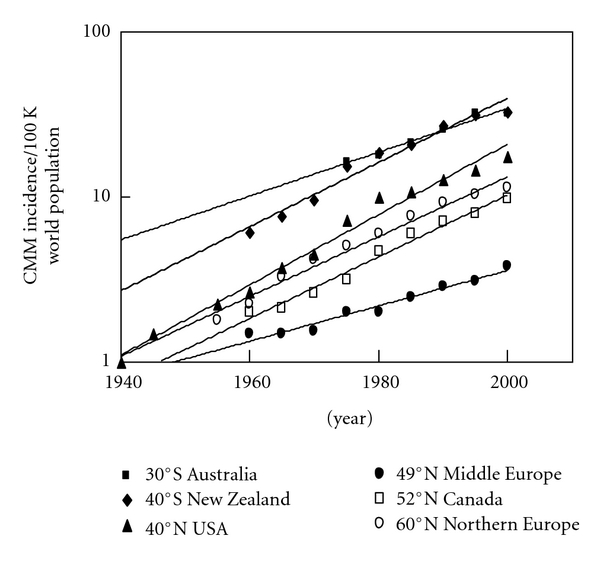
Temporal exponential increase in the incidence of CMM by latitude worldwide. Note that only New Zealand (40°S) and Northern Europe (60°N) have linear rates of increase.

One can obtain CMM incidence's throughout the decades from 1935 to 2007 for Australia, New Zealand, USA (except Connecticut 1935–1940 [1] and 1945 [2] and New York State 1955 [2]), middle Europe, Canada, and Northern Europe from the International Agency for Research on Cancer (IARC) [3–11]. The CMM incidence data from IARC was averaged from the following states/provinces/territories to get a mean value for each country at average latitude (Table 1 and Figure 1):

1. Australia (*∼*30°S; range 19.5–42.5°S)—Queensland (19.5°S), Western (24°S), South (32°S), New South Wales (33°S), Capital Territory (35.5°S), Victoria (36.5°S), and Tasmania (42.5°S).
2. New Zealand (*∼*40°S).
3. USA (*∼*40°N; range 20–47°N)—Hawaii (20°N), Los Angeles, California (34°N), Atlanta, Georgia (34°N), New Mexico (34°N), San Francisco, California (38°N), Utah (39°N), Connecticut (41.5°N), Iowa (42°N), Michigan (43.5°N), New York State (43°N; excludes New York City), and Washington State (47°N).
4. Middle Europe (*∼*49°N; range 46–52°N)—Switzerland (46°N), Slovenia (46°N), Romania (46°N), Hungary (47.5°N), Slovakia (48.5°N), France (48.5°N; Bas-Rhin), Germany (49.5°N; Saarland), all of Poland (51°N), The Netherlands (52°N), and all of England (52°N).
5. Canada (*∼*52°N; range 45–65°N)—Nova Scotia (45°N), New Brunswick (46.5°N), Prince Edward's Island (46.5°N), Ontario (51°N), Newfoundland (53°N), Quebec (53°N), Alberta (54°N), British Columbia (54°N), Manitoba (54°N), Saskatchewan (54°N), and Northwest Territories (65°N).
6. Northern Europe (*∼*60°N; range 53–65°N)—Ireland (53°N), Denmark (56°N), Scotland (57°N), Sweden (62°N), Iceland (63°N), Norway (64°N), and Finland (65°N).

**Figure 2:**
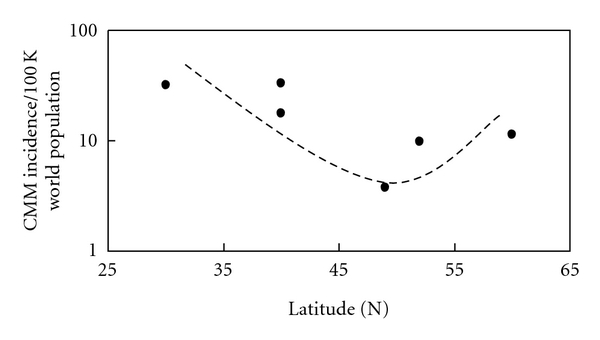
The incidence of CMM in the world's white populations for the year 2000 based on IARC data. The trend line indicates an increasing exponential incidence with decreasing latitude; however, a reversal appears to occur above *∼*50°N where the incidence increases with increasing latitude. Both Northern Europe and New Zealand, where there is ozone depletion, only have linear increases in the incidence of CMM; whereas, everywhere else in the world the increasing incidence is exponential. Note that in Northern Europe only Iceland has an exponential increase in CMM.

Figure 2 has the same CMM incidence data for the year 2000 as shown in Figure 1 only plotted by latitude [1–11].

**Figure 3:**
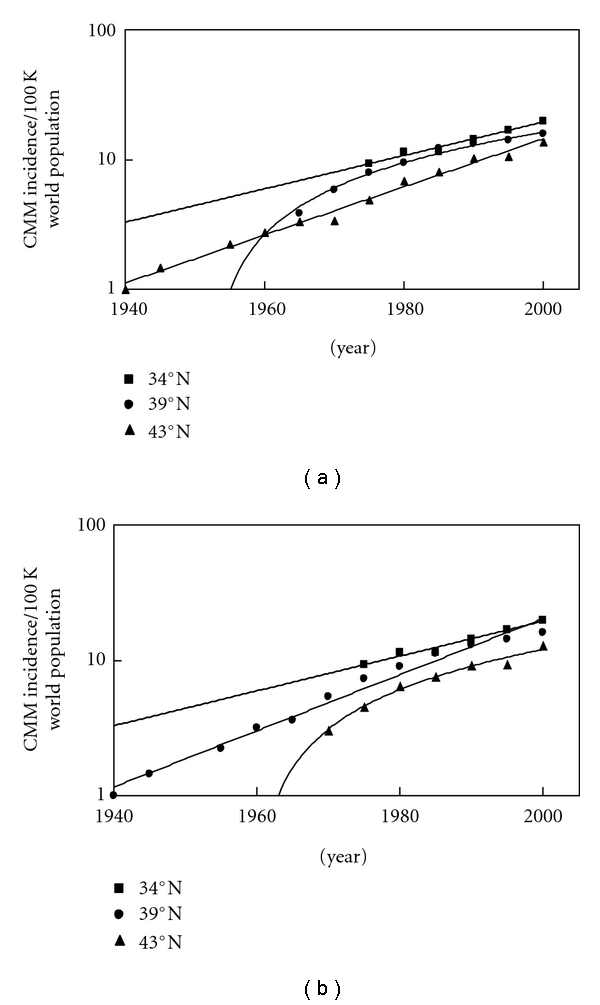
(a) The data indicates an increasing incidence of CMM in the white population of the USA for six decades at three latitudes: *∼*34°N (average of Los Angeles, California; Atlanta, Georgia; New Mexico), 39°N (average of San Francisco, California, and Utah) and 43°N (average of Iowa, Michigan, Connecticut, and Upstate New York). The CMM incidences at 34°N and 43°N are increasing at exponential rates while at 39°N, it is increasing at a linear rate (determined by comparing *R* ^2^ values for the linear and exponential trend lines). (b) The CMM incidence data at 39°N changes from linear to exponential and the 43°N data changes from exponential to linear when Connecticut is averaged with San Francisco and Utah.

**Figure 4:**
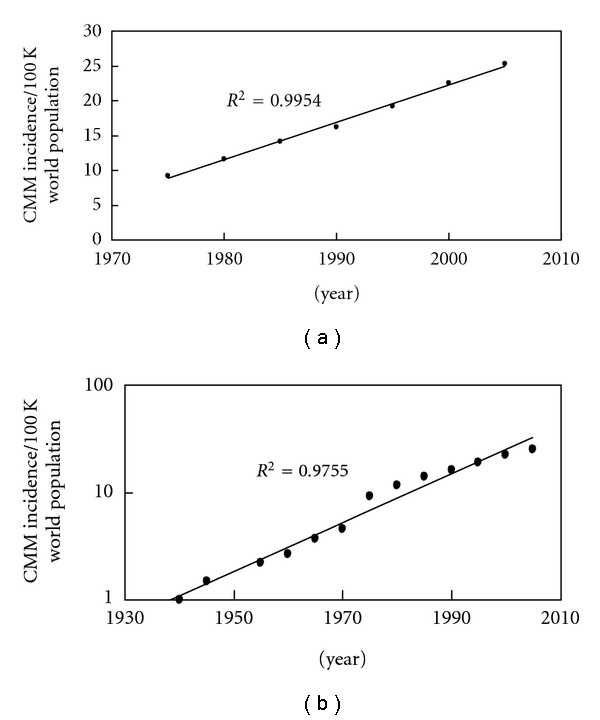
(a) The SEER 9 data from 1975 to 2007 indicates a linear increasing incidence in CMM (*R* ^2^ is 0.9954). Five years of data was averaged to obtain each data point, for example, the data point for 1975 includes averaged data from 1973 to 1977. If the data is plotted out year by year the trend line and *R* ^2^ values indicate it is a linear increase since 1973. Even if we add the IARC data for 1960, 1965, and 1970 to the SEER data, the incidence is still increasing in a linear manner from 1960 to 2005. (b) However, if one adds in the data for Connecticut and New York State extending it back 6 decades to 1940, then the increase appears to be exponential (*R* ^2^ is 0.9755) rather than linear (*R* ^2^ is now 0.9189).

Figures 3(a) and 3(b) show CMM incidence data from [1–11], which includes 10 registries, the same 9 registries as SEER 9 along with Los Angeles.  Figure 4(a) CMM incidence data for the USA (1973–2007) is from the SEER website at (http://seer.cancer.gov/faststats/selections.php) [12]. The data type is “SEER incidence,” the statistic type is “age-adjusted,” the year range is 1975–2007 (SEER 9), and the race/ethnicity is “white (includes Hispanics),” for both sexes of all ages. SEER 9 (white includes Hispanic) compared to SEER 17 (non-Hispanic white) has *∼*1-2/100,000 people lower incidence of CMM. The SEER 9 registries are Atlanta, Georgia; Connecticut; Detroit, Michigan; Hawaii; Iowa; New Mexico; San Francisco-Oakland, California; Seattle-Puget Sound, Washington; Utah. Data are available for cases diagnosed from 1973 and later for these registries with the exception of Seattle-Puget Sound and Atlanta. The Seattle-Puget Sound and Atlanta registries joined the SEER program in 1974 and 1975, respectively. Seer 11 includes Los Angeles and San Jose-Monterey, California starting in 1992.  Figure 4(b) shows the same data [12] as in Figure 4(a) only extended back to 1940 using data from [1–11].

## 3. Results

Table 1 contains the averaged CMM incidences of fair-skinned populations around the world from 1940 to 2000 for each country or region of the world [1–11]: Australia (~30°S), New Zealand (*∼*40°S), USA (*∼*40°N), Middle Europe (*∼*49°N), Canada (*∼*52°N), and Northern Europe (*∼*60°N). New Zealand has the highest incidence of CMM closely followed by Australia while Middle Europe has the lowest incidence.

Figure 1 shows a temporal plot of the CMM incidence from 1940 to 2000 [1–11]. Note that Australia, USA, Middle Europe, and Canada all have exponential increases except New Zealand and Northern Europe, which have linear increases. In fact, all the countries of Northern Europe have linear increases in CMM except Iceland, which is exponential.

Figure 2 shows the incidence of CMM in 2000 decreases with increasing latitude up to *∼*50°N where it changes and begins to increase with increasing latitude in Northern Europe [1–11]. Notice that the CMM incidence increases with decreasing latitude; however, near 50°N in Northern Europe the incidence begins to increase with increasing latitude. Note that the CMM incidence data from 1960 to 2000 all show the same change near 50°N (see Table 1). 

Figure 3 shows the CMM incidence data in the USA analyzed in different ways to know whether or not the increase is exponential or linear [1–11].  Figure 3(a) shows how the CMM incidence data at 39°N can be linear if we only average San Francisco and Utah together. The data at 43°N is exponential because Connecticut is included in that data set.  Figure 3(b) shows that when the Connecticut CMM incidence data is included with the 39°N data, it becomes exponential, while the data at 43°N becomes linear. Connecticut data extends a couple of decades further back in time (to 1935), which may be necessary to know if the trend is truly linear or exponential but might represent underestimates in CMM during that earlier time frame that would make the data appear to be exponential when it is really linear.

Figure 4(a) shows the SEER 9 data from 1975 to 2005 [12], which appears to be almost perfectly linear (*R* ^2^ is 0.9954), possibly because it does not extend far enough into the past as the data presented in Figures 1, 3(a), and 3(b).  Figure 4(b) shows the same SEER 9 data [12] in Figure 4(a) only extended back in time to 1940 using references [1] through [11]. This changes the USA data from a linear (*R* ^2^ is now 0.9189) to an exponential (*R* ^2^ is 0.9755) increase in the incidence of CMM.

## 4. Discussion

The incidences of CMM in fair-skinned, indoor-working people have been increasing worldwide for decades (Figure 1). The countries with the highest incidences per annual erythemally weighted UV dose [15] are closest to the equator: Australia (*∼*30°S), New Zealand (*∼*40°S), and the USA (*∼*40°N). The regions of the world with lowest fair-skinned incidences of CMM are in Middle Europe, around 49°N; however, the decreasing trend with increasing latitude changes around *∼*50°N so that Canada (*∼*52°N) and Northern Europe (*∼*60°N) have higher incidences than Middle Europe (Figure 2). In fact, Northern Europe at *∼*60°N has a higher incidence than Canada at 52°N and almost equates the incidence in the USA at *∼*40°N. The increasing incidence of CMM appears to be exponential in most regions except Europe; however, as analysis in Figures 3(a) and 3(b) show, data in the USA extending to 1940 can make the CMM incidence appear to be exponential. The SEER data from 1973 to 2007 suggests that the CMM incidence is linearly increasing in the USA (*R* ^2^ is 0.9954, Figure 4(a)). However, when the Connecticut [1, 2], New York State [2], and IARC data [3–11] extend the SEER data back to 1940, the incidence of CMM in the USA increases in an exponential manner (Figure 4(b)), possibly indicating that longer periods are needed to know if the increase is truly linear or if it is really exponential. Thus, we cannot be completely certain if the increase is linear or exponential in other countries because it may be that the data has to be collected for five decades or more to be conclusive.

Whether or not the incidence of CMM is increasing linearly or exponentially does not change the fact that it is increasing at the alarming rate of about 4-5% per year. In order to slow or stop this increasing trend, one must know what is causing it and change it. Based on the temporal plot shown in Figure 1, we know whatever started the increasing incidence of CMM either entered or left our environment before 1935, because that is when we have documented data for the first increases in CMM in the USA. Fluorescent lights (mid-1940's; [18]), sunscreens (late 1950's for UVB absorbing and 1988 for UVA and UVB absorbing; [19]), and tanning devices (*∼*1978; [20]) all entered our environment *after* the increasing incidence of CMM was first documented in the USA back in 1935 [1]. Thus, one should analyze what happened *before* 1935—during the early 20th century—to discover what may have really affected the incidence of CMM.

In the early 20th century, people went against evolution by going indoors during the day to work, which drastically decreased their daily amount of cutaneous vitamin D_3_ and exposed them to only UVA radiation passing through glass windows [14]. The artificial UV barrier created by windows divided UVB from UVA, so that the vitamin-D-making UVB wavelengths [21] were excluded and only the vitamin-D-breaking [22] and DNA-mutating UVA wavelengths [23–25] were included in our indoor-working environment. Possibly because this unnatural UV environment existed for decades in buildings and later in cars [26], CMM was promoted by UVA, after being initiated by UVB sunburns and began to steadily increase in the mid-1930's. 

Along these lines of reasoning, we now also have the increasing incidence of CMM with increasing latitude above *∼*50°N (Figure 2). People living above 50°N go to the beach during the summer and get sunburned at lower latitudes to initiate CMM and then return home to northern latitudes that have primarily UVA for most of the year to promote CMM. The higher latitudes also allow the sun to aim more often at a perpendicular angle to the window glass allowing more UVA to pass through and directly expose people's skin during their workday. In addition, above *∼*50°N there is little UVB to make cutaneous vitamin D_3_ most of the year [27]. Further, in the northern regions of the world (above 37°N), a vitamin D_3_ “winter” occurs from at least November to February, which extends from October to March at higher latitudes, when the dose rate of UVB is too low to make any previtamin D_3_ even if an office worker goes outside during peak hours [27]. On the other hand, UVB exposure during peak hours occurs to outdoor workers to some extent during their workweek, so that they can maintain adequate levels of vitamin D_3_ in their skin and blood (as 25-hydroxyvitamin D) for most of the year. Note that the blood levels of vitamin D (measured as 25-hydroxyvitamin D in serum) in outdoor workers (gardeners), who get about five times the solar dose that indoor workers get, are about twice as high as indoor workers [28]. The reason vitamin D is important for controlling melanoma is because it can be converted to the hormone calcitriol inside melanoma cells [29]. Calcitriol can control the growth [30–32] and apoptotic cell death [33] of responsive melanoma cells, while it also affects the immune system [34, 35] and inhibits tumor promotion [36], which may all be responsible for increasing the survival of melanoma patients who get regular, moderate sun exposures [37]. Thus, intermittent, strong UVB-induced sunburns may initiate CMM, while low concentrations of vitamin D_3_ [38] in the skin and UVA-induced DNA damage may promote CMM [14].

## 5. Conclusions

The incidence of CMM is increasing at an alarming rate around the world in fair-skinned, indoor-working populations, and may be increasing at an exponential rate. The CMM incidence decreases with increasing latitude up to ~50°N where it changes and increases with increasing latitude. This inverse may occur because there is more UVA relative to UVB for most of the year at higher latitudes compared to lower latitudes. If windows, allowing UVA to enter our indoor-working environment and cars, are at least partly responsible for the increasing incidence of CMM, then UV filters can be applied to office and car windows to help reduce the rate of increase in the incidence of CMM worldwide.

## Figures and Tables

**Table 1:** Averaged CMM age-adjusted incidences among whites per 100,000 people (world population) in each country of the northern and southern hemispheres (plotted in Figure 1); data is from [3–11] except 1940 [1], 1945, and 1955 [2] for the USA.

Latitude/place	1940	1945	1950	1955	1960	1965	1970	1975	1980	1985	1990	1995	2000
30°S Australia								16.33	17.89	21.43	25.90	32.20	32.13
40°S New Zealand					6.10	7.65	9.55	15.55	18.50	20.80	27.40	31.70	33.10
40°N USA	1.00	1.48		2.24	2.67	3.73	4.56	7.23	9.95	10.85	12.75	14.67	17.74
49°N Middle Europe					1.50	1.48	1.55	2.01	2.02	2.47	2.86	3.12	3.78
52°N Canada					2.00	2.12	2.64	3.18	4.73	5.97	7.08	7.95	9.74
60°N Northern Europe				1.80	2.24	3.28	4.18	5.09	5.99	7.68	9.20	10.29	11.33

## Abbreviations

CMM: Cutaneous malignant melanoma
IARC: International Agency for Research on Cancer
SEER: Surveillance epidemiology and end results
USA: United States of America
UVA: 321–400 nm
UVB: 290–320 nm.
